# “Well-Being in All Policies”: Promoting Cross-Sectoral Collaboration to Improve People’s Lives

**DOI:** 10.5888/pcd13.160155

**Published:** 2016-04-14

**Authors:** Thomas E. Kottke, Matt Stiefel, Nicolaas P. Pronk

**Affiliations:** Author Affiliations: Matt Stiefel, Center for Population Health, Kaiser Permanente Care Management Institute, Oakland, California; Nicolaas P. Pronk, HealthPartners, Minneapolis, Minnesota.

This article is a joint publication initiative between *Preventing Chronic Disease* and the National Academy of Medicine.

*The ultimate test of [health] policy is whether or not it adds to the well-being of the population served.*

Robert G. Evans and Gregory L. Stoddart (1)

In “A New Perspective on the Health of Canadians,” Marc Lalonde, the Canadian Minister of National Health and Welfare, concluded that health care does not have the power to fully mitigate the threats posed by unhealthful environments and behaviors (2). This 1974 report broke new ground by creating a comprehensive framework for the determinants of health based on 4 health fields — human biology, environment, lifestyle, and health care organization.

In 1990, perceiving that health *care* policy continued to dominate the formulation of health policy despite the Lalonde report, Robert G. Evans and Gregory L. Stoddart wrote “Producing Health, Consuming Health Care” (1). This landmark essay presented a series of progressively richer models that described the relationships among health, health care, the determinants of health, and well-being. They started with a model that they considered dominant at the time — a simple feedback loop between health care and disease as defined by the medical care system (Figure 1).

**Figure 1:**
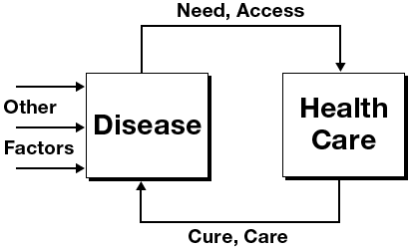
A model published by Evans and Stoddart (1) showing that health care was considered by many in 1990 to be the predominant determinant of disease. Reproduced with permission from Elsevier and G.L. Stoddart, 1990.

Regarding this model as too simplistic because it ignored the determinants of health identified in the Lalonde report (2), they also expanded the outcome measure progressively from the absence of disease as defined by the medical care system, to health and function as experienced by the individual, and finally to well-being, which they defined as the sense of life satisfaction of the individual. They postulated that a more complex model was a more accurate representation (Figure 2). 

**Figure 2:**
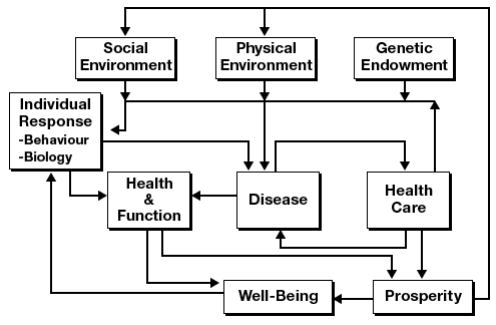
A model published by Evans and Stoddart (1) that accounted for multiple determinants of disease and health and function and defined well-being as the goal of policy. Reproduced with permission from Elsevier and G.L. Stoddart, 1990.

As did the World Health Organization (WHO) in 1948 (3), Evans and Stoddart viewed health as more than the absence of disease, but as the WHO did not, they explicitly distinguished health from well-being. They expressed the opinion that the WHO definition of health, “a state of complete physical, mental and social well-being and not merely the absence of disease or infirmity,” conflated health with well-being. Since then others have agreed. In a critique of the WHO definition in 1997, Rodolfo Saracci wrote, “Common existential problems — involving emotions, passions, personal values, and questions on the meaning of life — can make your days less than happy or even frankly uncomfortable, but they are not reducible to health problems” (4). Similarly, Christopher B. Forrest wrote in 2013 that the WHO definition “conflates health with happiness and life satisfaction, key dimensions of well-being” (5).

Evans and Stoddart wrote that well-being “is or should be (we postulate) the ultimate objective of health policy” and “[t]he ultimate test of [health] policy is whether or not it adds to the well-being of the population served.” However, they chose to focus their discussion on health, rather than well-being, as an outcome.

In 1986 the WHO Ottawa Charter for Health Promotion emphasized well-being as an end point, declaring that “[h]ealth is, therefore, seen as a resource for everyday life, not the objective of living” (6). Others have also framed health as an instrumental variable, as a means to the end of well-being (5). This perspective is consistent with that of contemporary social psychologists (7). Meanwhile, in health care circles, recognition of the importance of the social determinants of health is increasing, with health framed as the end goal, but recognition of the role of health as a means to the end of greater well-being is less well appreciated.

In 2003 Evans and Stoddart published a retrospective (8) on “Producing Health, Consuming Health Care.” Although they did find some cause for optimism, their frustration with the lack of interest in promoting the nonclinical determinants of health became clear when they quoted Homer Simpson: “Just because I don’t care doesn’t mean I don’t understand.” The United States does not seem to heed the message that the most significant determinants of health are not health care. Relative to other countries in the Organisation for Economic Co-operation and Development, a consortium of 34 countries dedicated to improving the economic and social well-being of people around the world, the United States continues a practice of overinvesting in health care and underinvesting in the other determinants of health (9). Between 1990 and 2014, health care spending in the United States increased from 12.1% to 17.5% of gross domestic product (GDP) (10). Despite this high level of investment, health outcomes declined relative to other developed countries during the same period (11).

## The Words We Use Influence Our Thinking

In the 19th century, linguists introduced the concept that language determines thinking (12). We believe that linguistic reasons explain why the broader determinants of health might not be taken into consideration when social policy is formulated in the United States. We wish to draw attention to 3 reasons in particular:

- Well-being is a positive concept. Despite all of the discussion that health is more than the absence of disease, the health metrics in current use are framed as the extent to which disease burdens the individual or the population. For example, disability-adjusted life years (DALYs) and quality-adjusted life years (QALYs) are defined as decrements from a year in perfect health; one of the most common measures of overall health in US national and state health surveys is the percentage of people with fair or poor self-reported health.
- The association of the word “health” with “health care” is so strong that it creates a conflation of “health care policy” with “health policy” that is impossible to break at times (1,13). This may be due in part to the size and powerful influence of the health care sector on public policy.
- In health care circles the expression “social determinants of health” is used frequently. Yet in educational or employment policy forums, the discussion is flipped to talk about the health determinants of educational attainment or productivity. Shifting the broad aim to well-being would appropriately place health among the determinants of well-being, as opposed to the ultimate aim. Policy makers, including those in health plans and care delivery organizations, may not recognize the nonclinical opportunities that they have at hand to improve well-being while staying true to their missions (14).

We believe that there is a way to mitigate these communication problems. Because “well-being” would simultaneously be a widely endorsed policy goal and a relatively empty space, we suggest that moving the policy discussion from health to well-being might be a way to negate the impact of conflating health care policy with health policy. A focus on well-being might also increase the willingness of policy makers in nonhealth sectors to join the challenge of improving health by addressing well-being. For individuals, opening the conversation with a discussion of their well-being goals might help them consider how their behaviors and environments contribute to or threaten their sustained well-being. Finally, a focus on well-being might help health policy makers recognize when their decisions will have a negative impact. For example, recognition is growing in Massachusetts that the increasing costs of health care have resulted in reduced spending for education, infrastructure, human services, and other public spending priorities that contribute to well-being (15).

Evans and Stoddart also stated in 1990 that “Our purpose is *not* to try to present a comprehensive, or even a sketchy, survey of the current evidence on the determinants of health. . . . Rather, we are trying to construct an analytic framework within which such evidence can be fitted” (1,16). Likewise, our goal for this essay is not to present a comprehensive framework for well-being as an end point of policy but rather to present a compelling enough argument that, if well-being is the end point, additional progress toward population health and well-being might occur. We therefore suggest, for the United States, the expression “well-being in all policies” be used instead of “health in all policies.” In the following paragraphs we present the evidence that supports this suggestion.

## Well-Being Is Not Just Physical Health

Although physical health and well-being are related, this relationship is much weaker than might be expected (17). The association between subjective health and life satisfaction is somewhat stronger but still far from unitary. For example, in a study based on nationally representative samples from the 32 countries that participated in the first 6 rounds of the European Social Survey, self-reported health ratings explained, on average, about 9% of the individual-level variance in life satisfaction; in no country did it explain more than 15% of the variance (18).

Subjective well-being is a broad category of phenomena that includes people’s emotional responses, levels of satisfaction in various domains, and global judgments of life satisfaction (17). It is not just the absence of mental illness; in fact, subjective well-being is a different psychological construct (19). Numerous scales have been created to measure subjective well-being, and these scales correlate to a great extent (17). “Flourishing,” a multicomponent construct that represents the state of complete mental health, is a widely accepted measure of subjective well-being (19). Although less robust than a multicomponent scale, both self-reported happiness and life satisfaction are also considered to be indicators of well-being (20).

## Well-Being Is Meaningful and Influential for Populations, Organizations, and Individuals

The Midlife in the United States (MIDUS) cohort follow-up study categorized participants as flourishing or languishing. Flourishing individuals reported the fewest health limitations of activities of daily living, the fewest missed days of work, the fewest half-day work cutbacks, and the healthiest psychosocial functioning (low levels of helplessness, clearly defined life goals, high levels of resilience, and high levels of intimacy) (19). After 10 years, the risk of death for individuals who were languishing was 60% higher than that for individuals who were flourishing (21).

## Well-Being Is Associated With Positive Social Policies

Evidence is clear that policies from diverse sectors — law, economics, public safety, and education, to name a few — affect well-being. Diener et al (22) observed that the happiest nations are economically developed and relatively wealthy, perhaps because the basic needs and desires of citizens are met to a larger extent in rich nations than in poorer nations. However, Diener et al also summarized the results of multiple studies listing several other modifiable characteristics of societies that have high levels of well-being. These societies have the following qualities:

- Strong rule of law and human rights
- Low rates of corruption
- Efficient and effective government
- Progressive taxation
- Income security programs, including adequate pensions, unemployment benefits, and support for the ill and disabled. They also have active public employment policies, including job training, employment incentives, and direct job creation.
- Political freedoms, with property rights, employment laws, and sound money
- Generous unemployment policies
- More healthful natural environments, for example, clean air and ample green space

Although the causes of a poor sense of well-being that lie in the physical or social environments — poverty, social isolation and exclusion, and unremitting stress, among others (23) — must be addressed if population-wide levels of well-being are to be significantly improved, individuals can improve their own well-being by practicing appreciation (24), gratitude (25), and kindness (26). It has also been observed that people who act happy tend to make other people happy (27).

## Momentum Is Building Toward Well-Being as a Policy Aim

Although the field of economics recognizes well-being as a goal (but has used the term “welfare” instead of “well-being”) (28), GDP has been the dominant measure of the prosperity of nations. However, there is a powerful movement away from using only economic indicators like GDP to represent prosperity and well-being in a population (20,29). Joseph E. Stiglitz, Amartya Sen, and others have advocated for well-being as a driver of social policy (30,31). National accounts of subjective well-being have been adopted in some form in more than 40 countries (22). In 2014 the Legatum Institute’s Commission on Wellbeing and Policy laid out the case for using well-being as the overall measure of prosperity and therefore as the yardstick for public policy (30).

Recognition is also growing at national policy levels of the benefits that accrue from greater integration of health care with social services to address the upstream determinants of health. For example, Finland has had a joint health and social services budget under the Ministry of Social Affairs and Health for many years (P. Puska, written communication, January 2016), and in 2009 Finland merged the National Public Health Institute of Finland and the National Research and Development Centre for Welfare and Health to form the National Institute for Health and Welfare. In 2014 the Scottish Parliament passed landmark legislation that “joined up” the health care and social services budgets (32).

In January 2016 the US Department of Health and Human Services announced the Accountable Health Communities Model. This funding opportunity focuses on linking clinical and community-based services that address a range of social needs, including transportation and housing (33).

In addition to merging health budgets and social services budgets, Finland created an initiative to expand the focus of health policy beyond health care policy (34). In contrast to the efforts of Evans and Stoddart to focus health policy on determinants other than health care, the Finnish initiative focuses on the health impact of policies formulated in sectors other than health, which they refer to as “health in all policies.” The goal is to ensure that the impact of all policies is to improve, or at least not threaten, public health and well-being. Considerable international experience in operationalizing the approach has accrued since Finland introduced it in 2006 (35).

## Opportunities to Improve Community Well-Being Exist Within the Missions of Both Public and Private Sectors

By their very nature, public sector organizations have an obligation to improve the well-being of the populations they serve. The focus of their activities include energy (clean, renewable energy vs polluting power sources), transportation (energy-efficient transit strategies that encourage active transport vs strategies dominated by private automobiles), community design (walkable, livable communities vs communities dominated by private automobile traffic), and education (early childhood education).

Evidence suggests that the private business sector can also do well by doing good. A recent report by the Vitality Institute connects integrated health and corporate social responsibility reporting with the “triple bottom line,” an accounting framework with 3 parts: social, environmental (or ecological), and financial (or economic) (36). Evidence that companies that intentionally create cultures of health, well-being, and safety are more profitable than their peer organizations is accumulating rapidly (37–40).

Because of the size of the health care sector (approaching a fifth of the US economy), the respected position of health care organizations in the communities they serve, the size of their physical plants, and their large number of employees, this sector has great potential to exert a positive impact on community well-being. However, not all leaders of health care organizations may recognize the benefits of broad-based initiatives or their opportunities to engage in them.

The following are examples of what Kaiser Permanente, HealthPartners, and selected other health care organizations are doing, and others could be doing, to improve community well-being.

**Kaiser Permanente.** The nation’s largest nonprofit integrated health system, Kaiser Permanente is advancing the concept of “total health,” an innovative framework focused on using all its assets to maximize physical, mental, and social well-being for its members and the communities it serves. To deliver on its total health ethos, Kaiser Permanente emphasizes using high-impact approaches such as workforce wellness initiatives for its employees and customers, increasing access to healthful foods and physical activity in thousands of schools, and reducing the organization’s institutional carbon footprint by purchasing green energy. To help drive local economic development in racial/ethnic minority communities across the country, Kaiser Permanente prioritizes supplier diversity, purchasing more than $1.5 billion from women- and minority-owned firms in 2014 alone (14,41).

**HealthPartners.** To promote its mission — to improve health and well-being in partnership with its members, patients, and community — HealthPartners adopted a community business model addressing nonclinical determinants of health in partnership with schools, foundations, nonprofits, and local and state government agencies (42). HealthPartners leaders are accountable to the board of directors for progress toward nonclinical goals just as they have traditionally been accountable for clinical care goals. Program examples include child-focused activities promoting healthful nutrition and physical activity (43–45), an advance care planning initiative to increase well-being at end of life (46), and a multisectoral campaign to eliminate stigma surrounding mental illness (47). HealthPartners is active in urban initiatives supporting education and health (48) and recently launched a 10-component Children’s Health Initiative with a goal of improving children’s health and well-being from birth through age 5 (49).

More examples of health plan programs that address the nonclinical determinants of health and well-being can be found at the Alliance of Community Health Plans (ACHP) website (50). ACHP recognizes the importance of taking a community-wide approach to improving health and well-being and describes these programs online as a resource for other organizations that wish to address the broad range of determinants of health and well-being.

## Closing Comments

Evans and Stoddart are only two of the many respected thinkers and political leaders who advocated for defining well-being as the ultimate goal of social policy after the Lalonde report was published. Adopting this convention could avoid the problems caused when health care policy is conflated with health policy. It may also increase the willingness of policy makers in all sectors to discuss how their policies add to or detract from the overall well-being of the individuals and populations they serve. Well-being is a widely endorsed concept and is associated with positive outcomes for individuals, organizations, and populations. Finally, it is measurable, modifiable, and influential. The words of Atul Gawande in *Being Mortal* (51) present a poignant description of why Americans would benefit from “well-being in all policies”:

We’ve been wrong about what our job is in medicine. We think our job is to ensure health and survival. But really it is larger than that. It is to enable well-being. And well-being is about the reasons one wishes to be alive.
